# Stimulated Ionic Telegraph Noise in Filamentary Memristive Devices

**DOI:** 10.1038/s41598-019-41497-3

**Published:** 2019-04-16

**Authors:** Stefano Brivio, Jacopo Frascaroli, Erika Covi, Sabina Spiga

**Affiliations:** CNR – IMM, Unit of Agrate Brianza, via C. Olivetti 2, 20864 Agrate Brianza, Italy

## Abstract

Random telegraph noise is a widely investigated phenomenon affecting the reliability of the reading operation of the class of memristive devices whose operation relies on formation and dissolution of conductive filaments. The trap and the release of electrons into and from defects surrounding the filament produce current fluctuations at low read voltages. In this work, telegraphic resistance variations are intentionally stimulated through pulse trains in HfO_2_-based memristive devices. The stimulated noise results from the re-arrangement of ionic defects constituting the filament responsible for the switching. Therefore, the stimulated noise has an ionic origin in contrast to the electronic nature of conventional telegraph noise. The stimulated noise is interpreted as raising from a dynamic equilibrium establishing from the tendencies of ionic drift and diffusion acting on the edges of conductive filament. We present a model that accounts for the observed increase of noise amplitude with the average device resistance. This work provides the demonstration and the physical foundation for the intentional stimulation of ionic telegraph noise which, on one hand, affects the programming operations performed with trains of identical pulses, as for neuromorphic computing, and on the other hand, it can open opportunities for applications relying on stochastic processes in nanoscaled devices.

## Introduction

Random telegraph noise (RTN) corresponds to random switching events of the flowing current that severely affect the reliability of nanoscale devices. The telegraphic current fluctuations are ascribed to phenomena of charge trapping and release into and from localized defects, which significantly influence the current conduction, e.g. by Coulomb repulsion, when confined to flow in nanometric cross-sections^1^.

In the last years, the investigation of RTN in resistive random access memories (RRAMs) and memristive devices has become a self-standing research field^2–7^. The most advanced memristive technologies rely on the voltage-driven formation and dissolution of a conductive filament (CF) shorting and disconnecting the two terminals of a metal/insulator/metal device^8^. The CFs are accumulations of defects that migrate at relatively high voltages and activate the current flow through a nanometric effective cross-section. Therefore, the charges trapped in defects in close proximity of the CF highly affect the current through the device, producing RTN^2–7^. RTN is usually investigated at a low static or quasi-static voltage, in which only the described electronic effects play a role. Indeed, high voltages are seldom employed for RTN investigation^6,9^, because they simultaneously produce both ionic re-arrangements and electronic charge trapping and de-trapping, resulting in deterministic non-volatile switching and random resistance fluctuations, respectively. The coexistence of the two phenomena at relatively high voltages prevents the disentangling of the effects of ionic migration, at the base of deterministic non-volatile switching, and charge trapping, leading to RTN. As a consequence, it is difficult to clearly identify ionic contributions to RTN, in addition to the electronic ones, though they can be expected^6,9^.

In this work, we demonstrate that random telegraphic current fluctuations can be intentionally stimulated by relatively high voltage pulses. Trains of pulses produce gradual resistance variation up to a saturation value. If the stimulation is carried on in such saturation region, no further neat resistance drift occurs and only the zero-average stimulated telegraph noise (STN) appears. In such saturation region, the two phenomena of random fluctuations and deterministic non-volatile switching are disentangled. The occurrence of both phenomena for the same voltage amplitude, when the pulses are first delivered and in the conductance saturation tail, suggests that the same physics could be involved. On the other hand, the pulsed stimulation of noise opens the unique possibility of investigating its dynamics, which qualitatively corresponds to that of the deterministic non-volatile switching. Since the non-volatile switching is universally recognized as produced by drift and diffusion of electrically active defects^8^, we ascribe the identified STN to ionic effects, as well. Furthermore, we demonstrate that the device resistance is the main parameter influencing the STN amplitude. We correctly describe the evolution of STN amplitude as a function of the average device resistance through a phenomenological filamentary model, in which ionic re-arrangements localized at the edges of the CF produce the observed current fluctuations.

It must be mentioned that, while conventional RTN influences the *reading operation* of the memristive devices, the described STN affects their *programming operation* when performed through trains of identical pulses. Such programming scheme is intensively investigated for applications in hardware neural networks. As a matter of fact, in a large number of publications^10–18^ also by other authors, the reported resistance evolution as a function of the number of identical pulses shows a large superimposed noise which is not contextually discussed and may be ascribed to STN. On the other side, we envision that the intentional activation of random resistance variations can be exploited for those applications relying on stochastic phenomena of nanoscaled devices, like random number generation^19–22^, physical unclonable function^23–25^, stochastic or chaos computing or emulation of stochastic cognitive neural processes^26–31^.

## Results

### Evidence of Stimulated Telegraph Noise

In filamentary memristive devices, the electric conduction through CFs with nanometric effective cross-sections is affected by the occasional trapping and release of charges into and from surrounding defects^2–7^. This phenomenon is particularly relevant for high resistance values in which the conductive path is partially interrupted or very thin^32^. In this manuscript, we demonstrate that telegraphic current fluctuations can be generated by ionic re-arrangement in HfO_2_-based memristive devices stimulated by voltage pulses, as described in the Methods Section.

**Figure 1:**
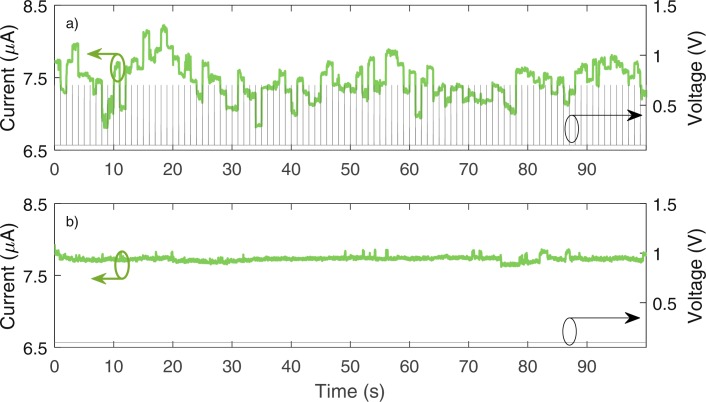
Evidence of Stimulated Telegraph Noise. (**a**) Current (green thick line, left axis) as a function of time in an experiment in which 100 pulses are delivered to the memristive device every 1 s. Pulses are 0.7 V high and 100 μs long and the current is acquired applying 0.1 V with a sampling time of 0.01 s. The applied voltage is reported as a grey thin line (right axis). Current jumps are ascribed to STN effect. (**b**) Current (green thick line, left axis) read at a constant voltage of 0.1 V (grey thin line, right axis) every 0.01 s with constant applied voltage and no pulses delivered to the device. Current jumps with small amplitude are ascribed to conventional electronic RTN.

Figure 1 reports a representative example of stimulated telegraph noise (STN) measured on a HfO_2_-based device programmed in a state with average resistance of about 13 *k*Ω. Concerning the data reported in Fig. 1a, 0.7 V pulses are delivered to the device every 1s (grey thin line in Fig. 1a right axis) and the current is read in between at a low voltage of 0.1 V (green thick line in Fig. 1a left axis). The current displays clear jumps in correspondence of the pulse delivery; in comparison the resistance value in between the pulses is more stable. The pulse parameters (pulse voltage and pulse time width) are chosen so that the fluctuations produced by the pulse trains average to zero and no appreciable switching is produced at the end of the measurement. Additional STN traces can be found in Supplementary Figs S1 and S2. Figure 1b reports the current acquired by applying the same reading voltage of 0.1 V without interleaving voltage pulses. Detected fluctuations have much lower amplitude (standard deviation of 33 nA) than those reported in Fig. 1a (standard deviation of 250 nA) and can be ascribed to conventional RTN. Figure 1b demonstrates also that the reading noise of our experimental apparatus is much smaller than the intrinsic device noise, in both cases of conventional RTN and STN. In summary, Fig. 1 demonstrates that voltage pulses can drive zero-average telegraphic current fluctuations. The resistance fluctuations stimulated by the high voltage pulses in Fig. 1a are stable for at least 1 s, which can only be ascribed to an ionic phenomenon. Indeed, according to related literature results, conventional RTN fluctuations, due to charge trapping and release, persist on average for few ms for the same reading voltages as for the present experiments^7,33,34^.

In the following, we refer to the average resistance value (i.e. the average in time of the signal affected by STN in Fig. 1a or equivalently the value in Fig. 1b with no pulse stimulation) as the *unperturbed* or *average* resistance value. Conversely, the resistance values that deviates from the average because of a stimulating voltage pulse are called *perturbed* resistance values. The *noise amplitude* is evaluated as the standard deviation of the measured resistance during pulse stimulation.

### Tuning noise with pulse parameters

**Figure 2:**
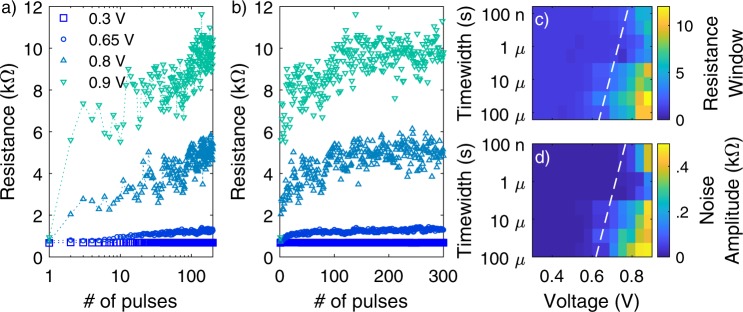
STN Characterization. Resistance as a function of the number of identical RESET pulses (**a**) logarithmic *x* axis, (**b**) linear *x* axis. Resistance is read at 0.1 V after each pulse. Series with pulses with different voltages and time width of 100 μs are displayed; final resistance window (*R*_*final*_/*R*_*initial*_) (**c**) and noise amplitude at resistance saturation (**d**) produced by sequences of 300 identical RESET pulses as a function of the pulse voltage and time width.

The pulse stimulation allows the noise characterization for different stimulus strengths (voltage and time width of the pulses). The immediate effect of the delivery of trains of identical pulses to an RRAM device is the change of its average resistance. Figure 2a,b reports the resistance dynamics of a device initialized in a low resistance state (resistance value of about 0.7–0.9 kΩ) and subjected to trains of identical pulses with various voltages from 0.3 to 0.9 V. Voltage polarity is chosen so as to increase the resistance (RESET operation). The resistance is read once after each pulse at 0.1 V. At the beginning of the pulse stimulation, the device resistance gradually drifts in an analogue fashion from low to high values, as evidenced in Fig. 2a with logarithmic *x* axis. In this regime, noise and non-volatile switching are superimposed. After a certain number of pulses, the resistance saturates around an almost constant average value which is perturbed by STN (evident in Fig. 2b with linear *x* axis). Therefore, STN is produced by pulses whose voltage is high enough also to promote the defect migration responsible for the neat gradual resistance change itself.

Resistance dynamics driven by trains of identical pulses as those shown in Fig. 2a,b have been widely reported in the recent literature papers and most of them show a superimposed significant noise in the RESET transitions, as well^10–18^. Since these works are mainly aimed at demonstrating gradual or analogue transitions of RRAMs devices, the noise is rarely discussed.

Figure 2b shows an apparent dependence of the noise amplitude on the pulse voltage (from pulse voltages of 0.3 V to 0.9 V for the same time width). However, the increase of the pulse voltage produces an increase of the average saturation resistance value. The unperturbed resistance value and the noise amplitude at the saturation are collected for several pulse voltages and time widths. Figure 2c reports the resistance window, defined as the ratio between the final saturation resistance and the initial value, as a function of the voltage and the time width of the stimulating pulses. The noise amplitude is evaluated as the standard deviation of the resistance values recorded over the last 100 pulses, where the random fluctuations average to zero. In this manner, the phenomenology of the switching is completely isolated from that of noise, which cannot be easily done with other characterization methods based on quasi static voltage ramps^6,9^. The map of the noise amplitude is reported in Fig. 2d and correlates very well with the trend of the resistance window, indicating that the average resistance value determines the amplitude of the STN. Furthermore, the maps of Fig. 2c,d can be used to identify the kinetics of the switching process and of the processes causing the noise, respectively. In particular, as a guide for the eye, white dashed lines are superimposed to the maps of Fig. 2c,d as an indication of the threshold voltage (vs time width) for the non-volatile switching and noise to occur, respectively. The matching of the both kinetics indicates that the origin of both phenomena must be ascribed to the same process of migration of ionic species.

Additional data for the resistance dynamics, resistance window and noise maps can be found in Supplementary Fig. S3. As shown in Supplementary Figs S4 and S5, the evolution of the STN in the SET process is less evident because high voltages bring the device to low resistance values. Therefore, voltages in principle high enough to stimulate noise are actually applied to low resistance device conditions where the noise is level is intrinsically low. Generally speaking, we can expect that the higher the average saturation resistance value in which a device can settle, the higher the STN amplitude. As a matter of principle, large and evident STN can be obtained also during SET operations if the saturation resistance value is sufficiently high. In addition, Supplementary Fig. S6 shows representative resistance evolutions driven by SET and RESET pulses as starting from similar initial resistance values. The higher STN amplitude shown by the RESET evolutions in comparison to those of the SET indicates that the occurrence of STN does not depend on the previous device programming; rather it is a further confirmation that the parameter governing the STN amplitude is the average resistance value.

### Noise stimulation at the dynamic equilibrium between drift and diffusion

In the previous paragraphs, we demonstrate the STN phenomena and its dependence on the resistance value of the device. We show that STN can be isolated from the non-volatile resistance switching in the saturation tails of the analogue resistance dynamics produced by trains of pulses and we provide further evidence that noise and non-volatile switching are caused by the same processes.

**Figure 3:**
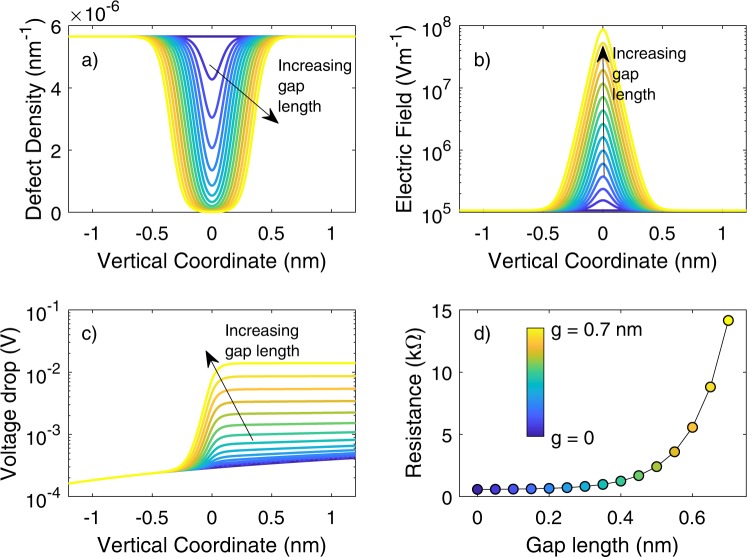
Model Evolution. Defect density (**a**), electric field (**b**) and voltage drop (**c**) as a function of the vertical coordinate for different values of the gap within a CF. (**d**) Device resistance as a function of the length of the gap within the CF. The colour-map for all the panels is reported in (**d**). The origin of the vertical coordinate axis is placed in the middle of the gap.

Filament formation and dissolution processes responsible for the non-volatile resistive switching are ascribed to drift (driven by electric field) and diffusion (driven by concentration gradient) of defects or ionic species that introduce electronic levels in between the forbidden gap of the insulating material. The description of the noise in the present manuscript derives from a previous modelling approach applied to HfO_2_-based device by the same authors^3^ and from other models reported in the literature^35,36^. We model the low resistance state of the device through a vertical cylindrical CF with radius of 3 nm connecting top and bottom electrodes. During a RESET process, an insulating gap is opened within the CF, increasing the device resistance. The electric conduction within the switching material is described through a defect density, *n*_*D*_, and a conductivity, *σ*^35,36^, that depends on the defect concentration:

1 $$\sigma ={\sigma }_{0}\cdot exp(-\frac{{E}_{a}}{{k}_{B}T}),$$

where *k*_*B*_ is the Boltzmann’s constant, *T* is the temperature, *E*_*a*_ is the activation energy for conduction and *σ*_0_ is a conductivity pre-factor. *E*_*a*_ is modelled according to the following equation^35,36^:

2 $${E}_{a}=\{\begin{array}{ll}{E}_{a,0}\cdot \{1-\frac{{n}_{D}}{{n}_{D},crit}\} & {n}_{D} < {n}_{D,crit}\\ 0 & \,{n}_{D}\ge {n}_{D,crit}\end{array},$$

where *E*_*a*,0_ = 0.01 eV, *n*_*D,crit*_ = 5.7 ⋅ 10^3^ m^−1^ is the critical defect density value above which the conduction becomes metallic. It is worth noticing that the CF evolves only in the vertical direction so that *n*_*D*_ can be considered as the defect density per unit length (along the vertical coordinate). The pre-factor of the conductivity per unit length *σ*_0_ is proportional to the defect density and varies linearly from 0.93 Ω^−1^m^−1^nm^2^ for *n*_*D*_ = 0 to 9 ⋅ 10^6^ Ω^−1^m^−1^nm^2^ ^35,36^. In summary, a CF shorting the two device electrodes is described as a high concentration of defects with cylindrical symmetry. We model the opening of a gap as a decrease of the defect density in the middle of the CF. First, we model the resistance as a function of the filament/gap structure. The different resistance states, in which the HfO_2_-based device stack can settle, result from a vertical conductive filament (CF) characterized by an interrupting gap with different length. Indeed, Fig. 3a shows the *n*_*D*_ profile along the CF for different gap lengths. The highest resistance state is associated to the largest (deepest) insulating gap within the CF (yellow line), while in the lowest resistance state the gap extension is almost negligible (blue line). For a constant reading current flowing through the device, the electric field along the CF axis is peaked on the centre of the gap and the larger the gap, the higher the peak, as shown in Fig. 3b. As a result, the voltage drop between the two terminals of the devices (along the CF) is higher, the larger the gap length, as shown in Fig. 3c. The resistance shown in Fig. 3d is the unperturbed value when voltages and currents are too low to produce any defect re-arrangement and it follows the trend reported in Fig. 3d as a function of the gap length.

**Figure 4:**
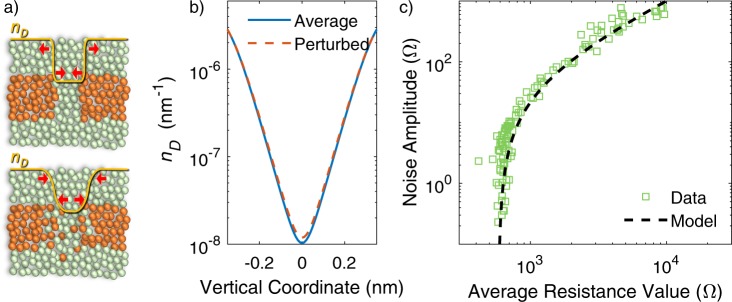
Comparison between data and model. (**a**) Sketch of the modification of the barrier edges during pulse stimulation. The *n*_*D*_ profile can be altered from a condition in which edges are sharp to one in which edges are smooth in correspondence of packed or slightly dispersed defects at the CF edges. (**b**) The *n*_*D*_ profiles, as used for the modelling in panel (c), as a function of the vertical coordinate in the average condition (straight line) and in the perturbed state (dashed line) zoomed around the gap/CF interface. The reported curves correspond to gap lengths of 0.7 nm. (**c**) Comparison of measured (symbols) and simulated (dashed line) noise amplitude as a function of the average resistance value.

In agreement with the picture of drift and diffusion of defects, we suppose that zero-average STN is the result of a dynamic equilibrium between the physical processes driving the resistance change itself ^37,38^. According to literature models, drift and diffusion combine to modify the concentration of defects, *n*_*D*_, inside the material according to the following equation:

3 $$\frac{\partial {n}_{D}}{\partial t}=\nabla \cdot (D\nabla {n}_{D}-\mu F{n}_{D}),$$

where *D* is the diffusion constant, *μ* the defect mobility and *F* the electric field. We assume that in a condition of dynamic equilibrium the diffusive term (first term on the right-hand side of equation (3)) balances the drift term (second term on the right-hand side of equation (3)). It is well known that the diffusion describes an entropic mechanism that tends to smooth out the concentration gradients. The diffusion is mainly important at the CF/gap interface, where the concentration gradients are the maximum. At equilibrium, the drift must counteract the smoothing action of diffusion. Therefore, we can model the effect of a pulse that produces the deviation of the device resistance from the average value as a change of the slope of the *n*_*D*_ profile at the CF/gap interface around the average (unperturbed) value. In particular, diffusion tends to reduce the slope of the *n*_*D*_ profile (reduce the gradient) and drift produces the opposite effect so that on average no net resistance change occurs. Figure 4a sketches the CF configuration in two representative cases of sharp and smooth profiles, which, in a pictorial view, can be associated to close packed and slightly dispersed defect arrangements. Orange balls represent electrically active defects. In case of a sharp profile of the CF edge, diffusion is predominant (Fig. 4a top), while in the case of smooth CF edge drift tends to re-establish a sharp CF edge (Fig. 4a bottom). A representative slight slope change is shown in Fig. 4b for a particular value of the gap length (=0.7 nm). The *n*_*D*_ profile, zoomed around one of the CF/gap interfaces, is plotted for the unperturbed (straight line) and for the perturbed (dashed line) cases. The slope change can be tuned to fit the experimental STN amplitude as a function of the unperturbed resistance value. The fit is shown in Fig. 4c and the fitting parameters are reported in Table 1. The good agreement justifies the model assumptions and indicates that the STN can be described through a phenomenological model that grasps a dynamic equilibrium of drift and diffusion tendencies acting on the CF/gap interface. Additional data on different sample align to the same experimental trend shown in Fig. 4c, as can be appreciated in Supplementary Fig. S7.

**Table 1:** List of model parameters and corresponding values.

Parameter	Value	Unit	Description
***I***	1	μA	Read Current
***r***	3	nm	Filament Radius
***g***_***min***_	0	nm	Minimum Gap Length
***g***_***max***_	0.7	nm	Maximum Gap Length
***n***_***D***, ***crit***_	5.6549e-06	nm^−1^	Critical Defect Concentration
***E***_***a***, **0**_	0.01	eV	Activation Energy for Electric Conduction
***σ***_**0**, ***min***_	0.93	nm^2^Ω^−1^m^−1^	*σ*_0_ at *n*_*D*_ = 0
***σ***_**0**, ***max***_	9.3 ∙ 10^6^	nm^2^Ω^−1^m^−1^	*σ*_0_ at *n*_*D*_ = *n*_*D*,*crit*_

## Discussion

In the present work, we demonstrate that ionic random telegraphic fluctuations of the resistance of filamentary memristive devices can be intentionally stimulated by pulses with suitable voltage levels. When a device in the low resistance state is stimulated by trains of identical RESET pulses, its resistance initially drifts in an analogue fashion towards high values. After a certain number of pulses, the resistance variation saturates and only random zero-average noise can be appreciated. In the saturation region, therefore, we can distinguish random resistance variations from the switching of the average resistance. The stimulated noise is telegraphic in the sense that voltage pulses trigger jumps in the resistance values that are afterward maintained at low read voltage until a new stimulus is provided. Noise is the result of voltage pulse stimulation because it does not occur in absence of pulses. The long retention times of the perturbed resistance values (measured up to 1 s) are only compatible with an ionic phenomenon, because any electronic process of trapping and release would result in a retention time for the perturbed state of few ms^7,33,34^. Furthermore, we analyse the kinetics of the processes activating the STN by varying pulse width and pulse voltage over a wide range of values. The qualitative agreement between the kinetics of processes producing non-volatile switching and STN indicates that both phenomena are driven by the same processes of drift and diffusion of defects. In agreement with all the experimental observations, we describe the STN as a dynamic equilibrium of the drift and diffusion tendencies at the CF/gap interfaces. Indeed, as described by Marchewka *et al*.^37^, during the RESET process, drift and diffusion approach an equilibrium configuration. In the present paper, we harness the dynamic nature of such equilibrium situation to generate telegraphic noise. On the contrary, the SET process of filamentary devices is typically self-accelerated due to thermal runaway^17,39,40^. This fact prevents the establishment of an equilibrium, which could be an additional factor hindering the identification of STN during SET transitions. On the other hand, it could be expected that, if the SET process is self-limiting, i.e. does not require external current limitation preventing device breakdown as in the present case, then the low resistance state is reached as an equilibrium between drift and diffusion. Therefore, as a matter of principle, in such case, telegraph noise can be stimulated during SET, provided the saturation resistance is sufficiently high.

The description of the switching in terms of gap and CF is purely phenomenological and descriptive. Indeed, a CF should be considered a region where defects reach such a high density that the localized states that they introduce in the forbidden energy band gap tend to interact and delocalize promoting electronic conduction in the formerly insulating material. Conversely, the insulating gap within the CF forms as a depletion (or de-activation)^41^ of defects in a region that was previously densely rich of them^3,42–45^. The proposed model intends to describe the noise within the same descriptive framework of CF and gap picture used to model the resistance switching and correctly describes the highlighted features of STN.

The present work, therefore, stands out from the previous literature in that random telegraphic variations are characterized in an un-explored voltage range and are ascribed to re-arrangement of defects constituting the CF responsible for the conduction in the oxide of memristive devices. As a matter of fact, usually conventional RTN is measured at low voltages where only electronic trapping and de-trapping processes can take place^3–5,33,46–52^. Only few works try to extend the characterization of RTN to quasi-static voltage ranges in which significant ionic contribution is present, but the simultaneous occurrence of noise and deterministic switching events makes their discrimination complex^6,9^.

Conventional electronic RTN has been the subject of wide research in the field of resistance switching devices, because it is an important source of noise affecting the *read-out* margin of RRAMs memory arrays^8^. We demonstrate here that ionic telegraphic variations appear also in the *programming* operation when it is operated through trains of identical pulses. Such programming scheme is specifically suitable for the expanding field of hardware neural networks in which a plastic response of memristive devices to repeated stimuli is desired. In particular, STN affects the ultimate resolution, i.e. number of distinguishable resistance levels, and the accuracy of a programmed resistance state of a filamentary memristive device. On the other hand, in most of the literature reports dealing with analogue plastic response of memristive devices, the switching dynamics is often associated with a large superimposed noise^10–18^, which is rarely discussed and which can be ascribed to STN.

On the other side, the intentional activation of random zero-average noise with tuneable amplitude as a function of pulse voltage and time width can open new opportunities for applications exploiting the inherent variability of memristive devices, such as random number generation, physical unclonable functions, stochastic learning systems and chaos and stochastic computing.

## Methods

### Device Fabrication

The experiments are performed on TiN/Ti/HfO_2_/TiN devices, whose operation relies on formation and dissolution of conductive filaments^32^. The device fabrication comprises sputtering deposition of the metal electrodes, atomic layer deposition of the amorphous oxide layer and patterning of the top electrodes by photo-lithography and lift-off, as specified elsewhere^2,3,12,32,53^.

### Electrical testing

A standard probe station equipped with Keysight B1500A instrument is used for device testing. Devices are electroformed with ramped current sweeps up to 1 mA. After forming, devices are ready for the switching operation. Pulses are sent through a B1525A Semiconductor Pulse Generator Unit and current is read through a High Resolution Source Measuring Unit (B1511B) both interfaced with the device through a custom board^54^. Voltage is applied to the TiN/Ti top electrode and the bottom TiN contact is grounded. Devices are subjected to trains of identical pulses and the resistance is read at 0.1 V. The pulsed operation can be performed without the need of any external element limiting the current, as discussed in refs^39,55^. Before the testing with pulses, the devices are initialized as described in the Supplementary Fig. S8.

## Supplementary information

Supplementary Information

## Data Availability

The datasets generated during and/or analysed during the current study are available from the corresponding author on reasonable request.
