# Supplementary material for: Stimulated Ionic Telegraph Noise in Filamentary Memristive Devices
Source: Sci Rep. 2019 Apr 16;9:6310. doi: 10.1038/s41598-019-41497-3 (PMC6465356; doi:10.1038/s41598-019-41497-3)
Supplement: Supplementary file 1 — Supplementary Information [file 41598_2019_41497_MOESM1_ESM.docx]

Supplementary Information

Stimulated Ionic Telegraph Noise in Filamentary Memristive Devices

Stefano Brivio*, Jacopo Frascaroli, Erika Covi and Sabina Spiga*

CNR – IMM, Unit of Agrate Brianza, via C. Olivetti 2, 20864 Agrate Brianza, Italy
*E-mail: stefano.brivio@mdm.imm.cnr.it, sabina.spiga@mdm.imm.cnr.it

**Representative STN traces**

**Figures S1** and **S2** report additional STN noise traces stimulated by trains of $10$ and $50$ voltage pulses, respectively. Each pulse drives a sharp current jump. The current level is maintained until the arrival of the next pulse.


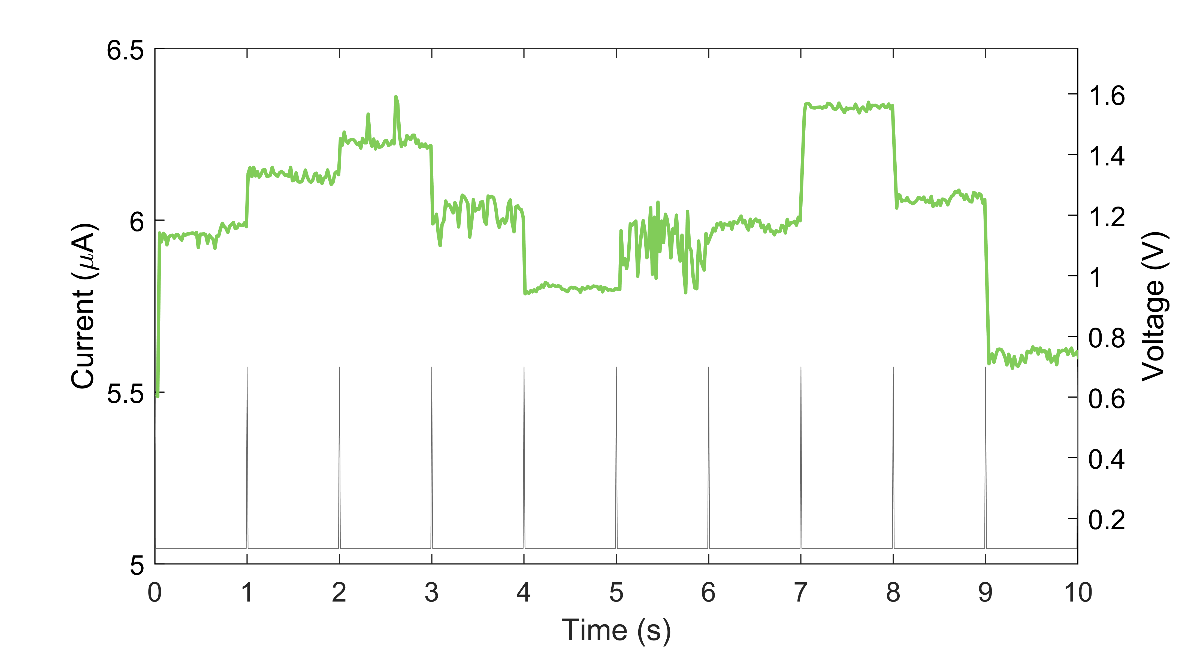


***Figure S1.*** *STN current traces under stimulation with* $10$ *pulses with voltage of* $0.7$ *V and* $100 \mu$*s width.*


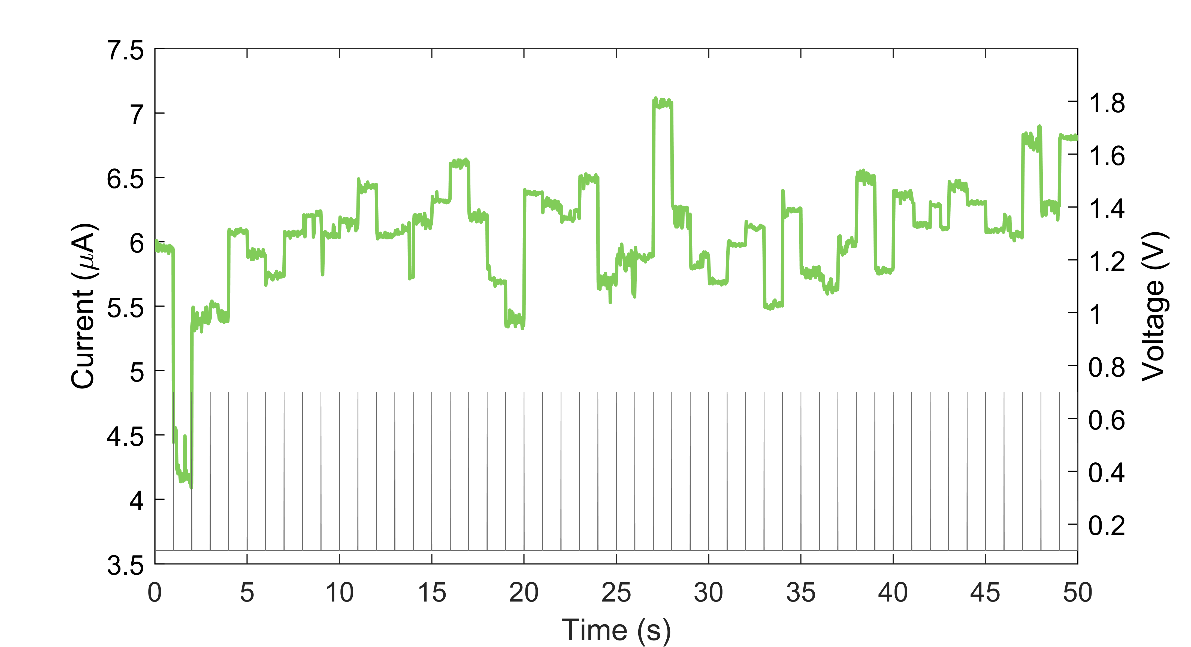


***Figure S2.*** *STN current traces under stimulation with 50 pulses with voltage of* $0.7$ *V and* $100 \mu$*s width.*

**Further STN characterization**

We report here the noise analysis performed on an additional sample operated in a lower resistance range. The device analyzed in the manuscript will be called *sample A* and the device, whose results are shown only in this supporting material, will be called *sample B*.

**Figure S3** reports STN characterization as a function of RESET pulse parameters for a *sample B*.


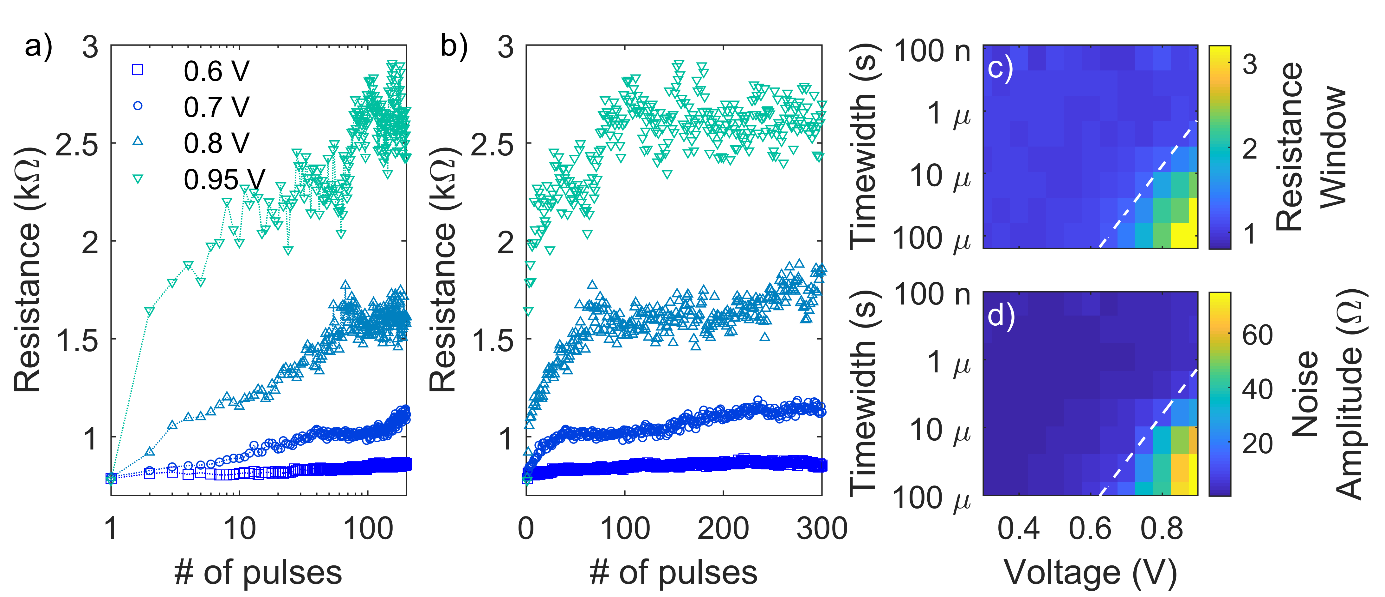


***Figure S3.*** *Resistance as a function of the number of identical RESET pulses (a) logarithmic x axis, b) linear x axis). Resistance is read at low voltage after each pulse. Series with pulses with different voltages and time width of* $100 \mu$*s are displayed; final resistance window (*${R_{final}}/{R_{initial}}$*)(c) and noise amplitude at resistance saturation (d) produced by sequences of* $300$*identical RESET pulses as a function of the pulse voltage and time width. Sample B.*

The characterization of the noise for the SET dynamics is reported in **Figures S4** and **S5** for samples A and B, respectively. The SET transition is rather abrupt with respect to the smoothness of the RESET operation as visible by the large resistance jumps in **Figure S4a** for voltage pulses strong enough to promote the resistance transition. The resistance dynamics also shows that there is no precise correlation between the resistance window (**Figures S4b** and **S5b**) and the standard deviation of the resistance in the last $100$ pulses (i.e. the noise amplitude in **Figures S4c** and **S5c**). Indeed, for *sample A*, the noise amplitude is low for intermediate resistance windows (please compare Figures S4b and S4c), while for *sample B* the complementary situation is obtained (please compare Figures S5b and S5c). In summary for the SET operation, it is difficult to isolate the STN phenomenology. The unclear noise phenomenology is ascribed to the fact that voltage pulses strong enough to produce noise actually as a first effect bring the device in the low resistance state, where the noise amplitude is intrinsically small, according to the Figure 4 of the manuscript.


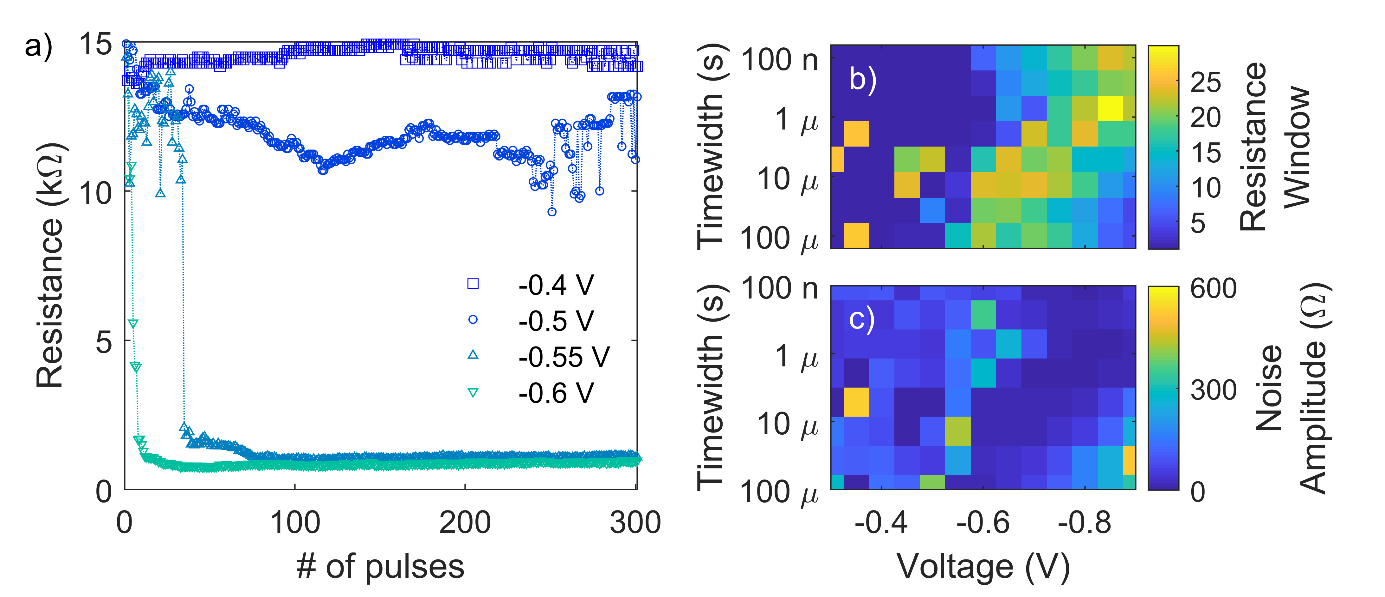
***Figure S4.*** *Resistance as a function of the number of identical SET pulses (a) logarithmic x axis, b) linear x axis). Resistance is read at low voltage (100 mV) after each pulse. Series with pulses with different voltages and time width of* $100 \mu s$*are displayed; final resistance window (*${R_{final}}/{R_{initial}}$*) (c) and noise amplitude at resistance saturation (d) produced by sequences of* $100 \mu s$ *identical RESET pulses as a function of the pulse voltage and time width. Sample A.*


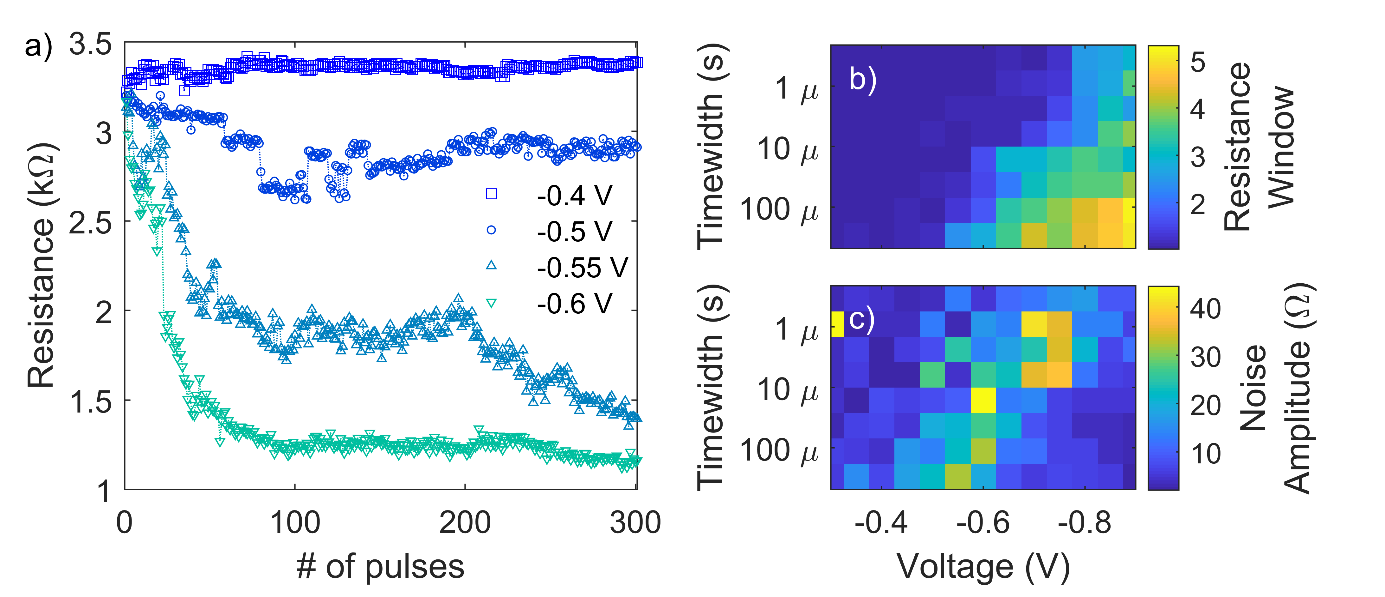


***Figure S5.*** *Resistance as a function of the number of identical SET pulses (a) logarithmic x axis, b) linear x axis). Resistance is read at low voltage after each pulse. Series with pulses with different voltages and time width of* $100 \mu s$ *are displayed; final resistance window (*${R_{final}}/{R_{initial}}$*) (c) and noise amplitude at resistance saturation (d) produced by sequences of 3*$00$*identical RESET pulses as a function of the pulse voltage and time width. Sample B.*

In order to exclude that the different STN behavior obtained through train of SET and RESET pulses, we record the noise traces during SET and RESET (RST) operation starting from similar resistance values but different pulse stimulation history (we performed sequences of 1 SET and 1 RESET operations). The SET and RESET traces are reported in **Figure S6a**. Variability exist among the set of curves as already discussed in previous work.^1^ It is evident that STN is larger for RESET operation than for SET operation. As a further prove, the resistance fluctuations in the last 100 delivered pulses are compared **in Figure S6b** for SET and RESET operation.

***Figure S6.*** *a) SET and RESET (RST) operation driven by train of pulses starting from initial states with similar resistance values but different programming history. The used voltages are* $-0.8 V$ *for SET and* $1 V$ *for RESET and the timewidths of the pulses is* $30 \mu s$ *for both operations. b) resistance fluctuations in the last* $100$ *pulses for SET and RESET (RST) operations.*

**Comparison between Data and Model**

**Figure S**7 reports the comparison of simulation and the experimental data for the RESET operation of *samples A* and *B* of the noise amplitude as a function of the average resistance value.


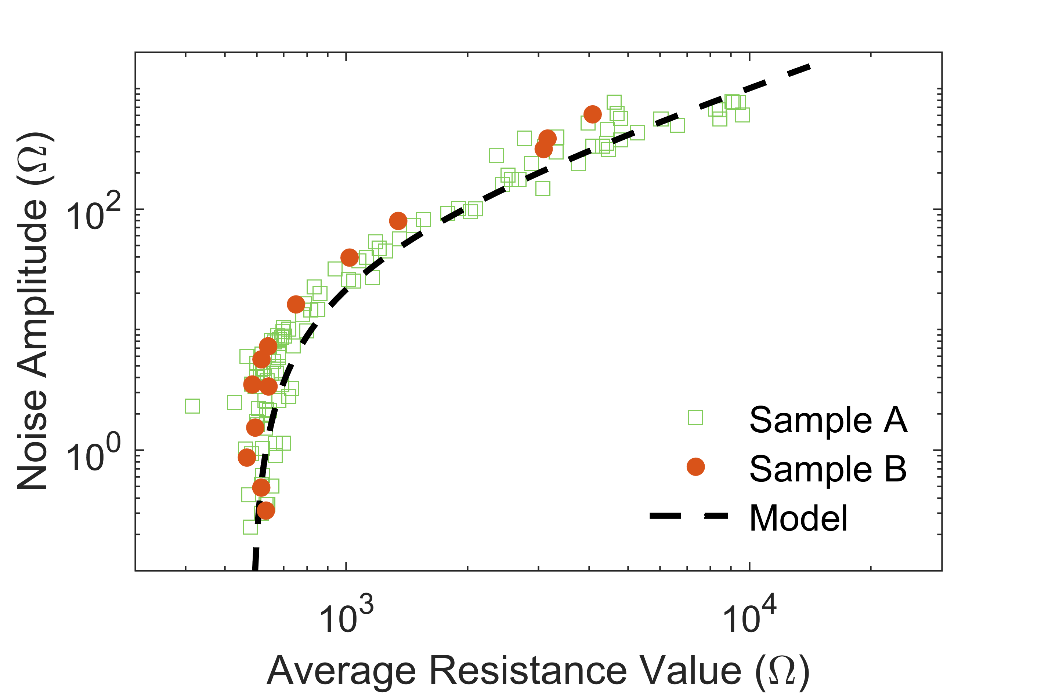


***Figure S7.*** *Comparison of measured (symbols) and simulated (dashed line) noise amplitude as a function of the average resistance value. The shown experimental data are measured on sample A and B.*

**Device Initialization**

As prepared fresh devices require an electroforming step to initiate the reversible switching operation. A representative electroforming sweep is shown in **Figure S8a**, in which the current is swept from ${10}^{-9} A$ to $3\cdot{10}^{-4} A$ and the voltage is measured. In current controlled sweeps, the switching to low resistances is visible and a nearly horizontal transition. After the electroforming, the device shows Complementary Resistive Switching (CRS) operation in which for each voltage polarity a SET operation and a RESET operation occurs in succession as shown by the 1^st^ and the 2^nd^ operations in **Figure S8b**. The CRS operation occur as a results of the symmetry of the electronic and ionic responses of the opposite interfaces of the device.^2,3^ Once a compliance current is set, the usual bipolar operation is reached through a SET operation (3^rd^ operation in **Figure S8b**). Before the pulse testing, the repeatability of the bipolar operation is checked over 20 RESET (RST) and SET cycles as those shown in grey in **Figure S8b**.

**

***Figure S8.*** *a) forming operation in current controlled mode. b) device initialization consisting in 2 CRS loops, a SET operation and cycling over 20 RST and SET cycles.*

**References:**

1. Frascaroli, J., Brivio, S., Covi, E. & Spiga, S. Evidence of soft bound behaviour in analogue memristive devices for neuromorphic computing. *Scientific Reports* **8**, 7178 (2018).

2. Covi, E. *et al.* Analog Memristive Synapse in Spiking Networks Implementing Unsupervised Learning. *Front. Neurosci* **10**, 482 (2016).

3. Brivio, S. *et al.* Experimental study of gradual/abrupt dynamics of HfO_2_-based memristive devices. *Applied Physics Letters* **109**, 133504 (2016).
